# Utilizing the Nursing Professional Development Model to create and sustain nursing education aimed at improving the care of patients with Parkinson’s Disease in the hospital

**DOI:** 10.3389/fmed.2023.1275970

**Published:** 2024-01-24

**Authors:** Mary Bobek, Pamela Pascarelli, Lisa Cocoziello, Hooman Azmi

**Affiliations:** ^1^Department of Clinical Education, Hackensack University Medical Center, Hackensack, NJ, United States; ^2^Department of Neurosurgery, Hackensack University Medical Center, Hackensack, NJ, United States; ^3^Hackensack Meridian School of Medicine, Nutley, NJ, United States

**Keywords:** nursing education, medication, safety, Parkinson’s Disease, hospitalization, Nursing Professional Development Model

## Abstract

The Nurse Professional Development Model (NPD) has been utilized to improve quality of care for several conditions. Patients with Parkinson’s Disease (PD) are susceptible to higher risks while in the hospital. Educational efforts for this patient population are challenged by the small, disbursed number of patients as well as increased turn-over and reliance on temporary nursing staff. To properly care for this patient group, any education has to be hospital wide and ongoing for maintenance of competency. We have used the NPD Model to initiate education for new incoming nurses as well as for continued education for a program that requires hospital-wide reach. Our utilization of the NPD Model for this high risk, low volume patient population has helped us improve the safety of this patient population in the hospital. With this manuscript we detail the need and the educational platform with the hope of it serving as a reference for other institutions facing similar challenges.

## Introduction

Patients with Parkinson’s disease (PD) often rely on strict medication regimens to help control their symptoms. Motor fluctuations as well as troubling dyskinesias may develop over time requiring exact timing and doses of medications to maintain mobility and quality of life. Delays or omissions or receipt of inappropriate medication can have a profound negative impact on this patient population (1–3). Approximately 75% of patients with PD admitted to the hospital do not receive their medications on time, (4) and of these, over 60% will experience complications that can be harmful, increase lengths of stays or even increase risk of mortality (5). Compounding the issue is the potential administration of some commonly used medications which can be harmful for PD patients (6). Parkinson’s Disease patients can be admitted to any adult in-patient unit and often PD is not the admitting diagnosis (7). Deficiencies in knowledge about the care of PD patients among healthcare providers compounds these issues (8).

Each year over 300,000 PD patients are admitted to US hospitals (9). While this is overall a large number, each of the estimated 6,000 US hospitals may receive a much smaller proportion. The relatively small number of patients scattered throughout the hospital presents a challenge in the development and maintenance of competency for the care of this patient population. Lack of national standards and guidelines for the care of PD patients in the hospital also adds to the obstacles. We have developed a program at our institution centered on ensuring adherence to a patients’ home medication regimen when placing orders for PD medications, based on recommendations from the Parkinson’s Foundation, which in response to a crisis brought forth by patients and caregivers of dangers of hospitalization, has been advocating for awareness in this arena and for development of safety measures for those with PD in the hospital (5). Our program has had some success, and we have seen a reduction in the length of stay of this patient population after implementation (10, 11). We have utilized the Nursing Professional Development (NPD) Model to educate and to maintain competency for our nursing staff and ensure adherence to the protocol amidst nursing shortages and high turnover rates which are industry wide (12). We are presenting our experience in the use of the NPD model for hospital wide education and maintenance of competency.

Parkinson’s Disease Patients are considered a “high-risk, low frequency” patient population as compared to other hospital-wide diagnoses. While 16–45% of PD patients will visit an Emergency Department once a year and 7–28% of these patients will be hospitalized (5). In comparison to diagnoses such as heart failure, PD frequency in the hospital is relatively smaller. From the year 2019–2022 we had a total of 1,517 patients admitted to our hospital who had a diagnosis of PD as a primary or non-primary diagnosis (annual average 379 patients). As a comparison during the same time period, 3,674 patients were admitted for heart failure (average annual admissions 918 patients). Likewise, a primary or secondary Diabetes diagnosis averages 700–800 admissions *per month*. Because of the lower number of annual admissions, nursing staff will encounter a PD patient less frequently compared to other chronic disease processes during an average nursing shift. With more than half of PD patients experiencing a complication while in the hospital (2, 5), nursing education and heightened awareness is crucial in supporting patient care and optimal patient outcomes. This creates an educational challenge for standardizing initial nursing education as well as maintaining competency to ensure nurses can recognize a patient with Parkinson’s Disease and care for this high-risk population.

Complicating the matter further, the nursing landscape has shifted nationally, and our institution has not been immune to these changes. Over the past 6 years, the number of new graduate & temporary nurses hired has consistently increased annually (Table 1) as the number of experienced nurses hired has decreased.

**Table 1:** Number of new RNs hired per year, level of experience, and temporary status.

Year	New Graduates	Experienced	Temporary nurses
2016	54	176	33
2017	83	223	65
2018	112	147	43
2019	123	153	61
2020	*	*	*
2021	155	125	192
2022	223	294	192

*Pandemic year, data not collected.

## Education opportunity

This high-risk, low frequency population, combined with a less-experienced nursing workforce, has created a significant opportunity for education related to Parkinson’s disease. This education included the importance of medication timing and associated complications. PD patients are admitted hospital wide based on their admitting diagnoses; therefore education must extend to all adult inpatient nursing units.

## The nurse educator role

Nursing professional development (NPD) is a specialty of nursing practice which is defined by standards, based on research, and critical to quality patient and organizational outcomes (13). This specialty improves the professional practice and role competence of nurses and healthcare personnel by facilitating ongoing learning, change, and role competence and growth with the intention of improving population health through indirect care (14). While this model is more adopted in the US, there are other similar models internationally (15, 16).

The Association for Nursing Professional Development (ANPD) has defined the qualifications of NPD practice. There are two levels of practice which are determined based on degree (Bachelor of Science in Nursing, Master of Science in Nursing, Doctorate) and presence of a national NPD certification. There is evidence of the impact of NPD practitioners (“nurse educator”) in an organization and the effect on nursing orientation time, nurse turnover rates, retention, patient satisfaction and organizational outcomes. To assess the impact of NPD practitioners on unplanned hospital visits, Harper, Maloney, Aucoin & MacDonald (17) conducted a replication study of NPD practitioners from 398 participant responses from organizations in 46 states in the US and District of Columbia. Included were those with capacities from 200 beds up to 4,000 beds, stand alone and network systems, profit, non-profit and all levels of care. The study observed that higher numbers of NPD practitioners per hospital were associated with statistically significant (*p* ≤ 0.10) lower unplanned visits for heart failure, pneumonia and CABG.

## Nursing Professional Development Model

**Figure 1:**
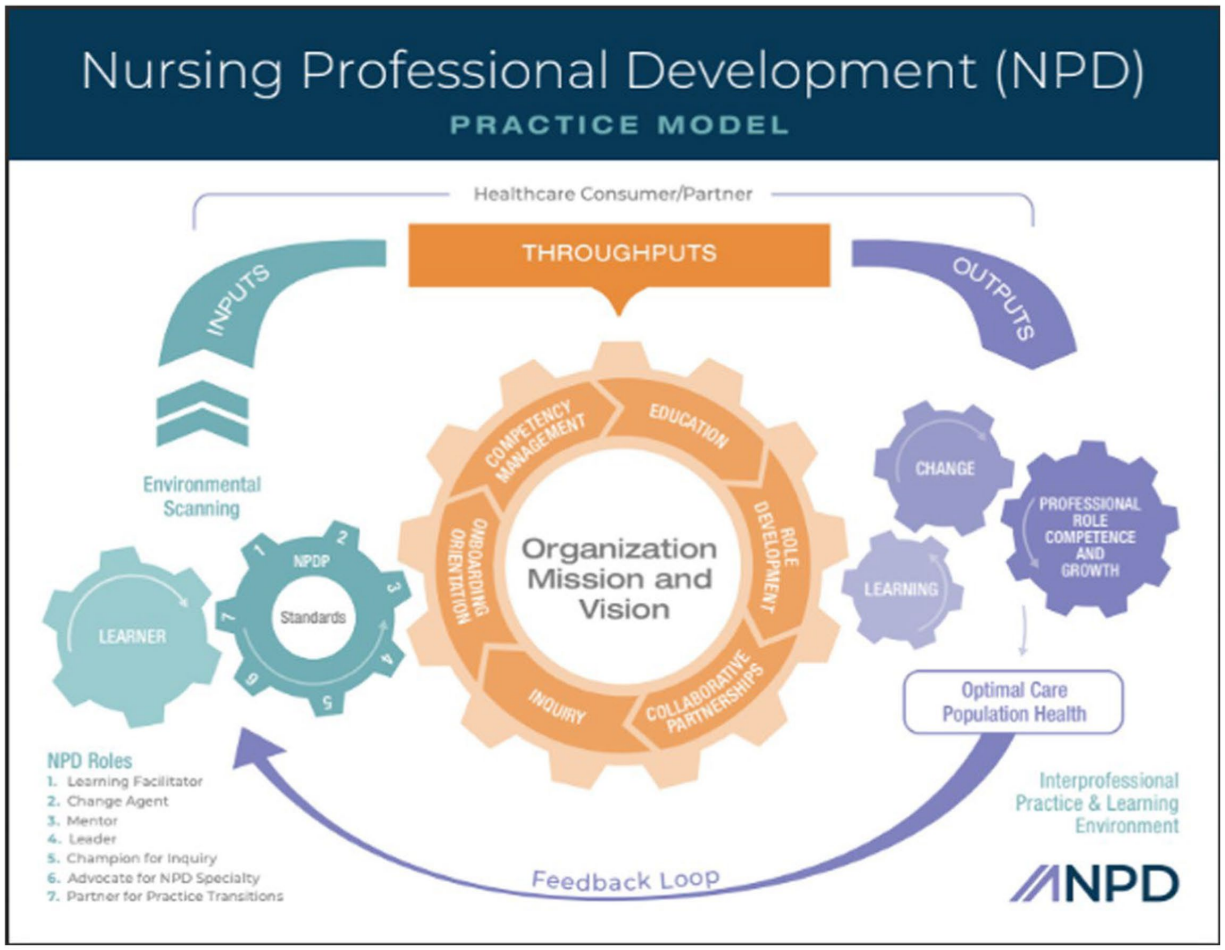
Nursing Professional Development Practice Model.

ANPD has defined the scope of practice which is depicted in the NPD Practice Model. This model (Figure 1) is defined by inputs, throughputs and outputs. The model begins with environmental scanning (the assessment) which is a process of systematically monitoring an organization’s internal and external environments for earliest signs of opportunities and challenges that may create or reveal a potential professional practice gap (14). The inputs delineate the “when, who and how” in the scope of practice. NPD practice is a continuous assessment of educational opportunities or gaps in practice. The learner and NPD practitioner interact with the goal of impacting measurable outcomes. The model further defines the roles of an NPD practitioner which are learning facilitator, change agent, mentor, leader, champion for inquiry, advocate for the specialty and partner for practice transitions. The throughputs are the processes that occur in the interprofessional learning environments (clinical, classroom & online) and are facilitated by the NPD practitioner. The throughputs represent the responsibilities of the NPD practitioner or the “what” of the NPD scope of practice and are guided by the “why” which is the organization mission and vision. The major responsibilities are onboarding/orientation, competency management, education, role development, collaborative partnerships and inquiry. Lastly the outputs represent the overall desired outcomes of the NPD practice that align with the mission and vision. Expected outcomes surround learning, change, professional role competence and growth which leads to optimal care in population health (14).

## Applying the model to practice

### Inputs

Through the process of environmental scanning, we identified nursing education needs related to the changing nursing landscape. As a result of the increase in newly hired nurses, initial education was needed as well as a means to maintain competency caring for this high-risk, but low frequency population. Additionally, we assessed issues that can put PD patients at risk during their hospitalization such as adherence to medication timing and promoting early mobility.

### Throughputs: onboarding

All new hires onboarded to our institution attend a full-day course related to clinical practice guidelines for various diseases which includes a presentation on Parkinson’s Disease. This class is an introduction to the high standards we hold for patient care and is where nurses receive their initial PD education. This one-hour presentation includes: an overview of PD, nursing assessments and care of the PD patient within the nursing process, common PD medications, contraindicated medications, importance of medication timing, surgical treatment, safety concerns, and the nursing care plan.

### Throughputs: education

Education is provided through multimodal platforms. An annual online Parkinson’s Disease specific module in our electronic learning management system is assigned to every adult inpatient nurse. This module covers pathophysiology of Parkinson’s Disease, the importance of medication timing, contraindicated medications for PD patients, and the importance of early mobility. This education is provided to the nursing team as well as other members of the interdisciplinary team which include Physical Therapy, Occupational Therapy, Patient Care Technicians, Nursing assistants, Dietary, and Pharmacy. This module can be considered initial education and ongoing competency education for the interdisciplinary team.

Nurses on high volume units such as the neuroscience areas receive additional education. An initial education class was developed for a new medical/surgical neuroscience unit at our institution. It incorporated a one-hour presentation for care of Parkinson’s Disease patients. Our Neurosurgical Intermediate Care Unit receives a unit-specific course, taught by nursing peers, which includes a discussion on Parkinson’s Disease.

Early mobility initiatives were implemented for all patient populations which further supported the care of PD patients to prevent deterioration of motor symptoms. To help improve mobility, the Bedside Mobility Assessment Tool (BMAT) was implemented in 2016 as well as a “Move to Improve” initiative in 2021 which was a hands-on class to support nursing staff with mobility tools. In 2017 educational webinars were developed with a pre & post assessment for nurses on mobile devices as an additional approach to education. Table 2 summarizes the educational components.

**Table 2:** Throughputs: educational components.

Educational component	Throughputs	Initial education and competency management	Target audience	Teaching method
PD Presentation for newly hired nurses	Onboarding	Initial	All nurses	Classroom
Neurosurgical intermediate care class	Education	Initial & competency	Intermediate care neuroscience nurses	Classroom
Neuroscience day class	Education	Initial	Neuroscience nurses	Classroom
Annual learning module	Competency management	Competency	All nurses	Online
Move to improve	Education	Initial	All nurses	Classroom
Annual competency class	Competency management	Competency	All nurses	Classroom & online
Unit nurse champions	Role development	Competency	All nurses	On unit support
Time critical medical alert	Inquiry	Competency	All nurses	Electronic medical record (EMR) alert tool
Nursing care plan	Collaborative partnerships	Competency	All nurses	EMR real-time best practice guidance

### Throughputs: competency management

Maintenance of clinical competency when caring for patients with Parkinson’s disease is crucial. Competency is an already expected level of performance that integrates knowledge, skills, and judgment and is considered an outcome and an ongoing process (14). In addition to what was described above, we have created additional methods for maintaining competency.

PD case studies are utilized in our annual adult in-patient nursing competency class. From a safety perspective, laminated posters have been created and displayed near the medication dispensing systems reminding nurses of the contraindicated medication for PD patients. NPD practitioners and a neurosurgery advanced practice nurse also collaborate on unit-based in-services to support competency and raise awareness (Table 2).

Additionally, we have created a Parkinson’s Disease awareness “banner” which is an identification tool to alert all health care team members that they are caring for a PD patient regardless of the admitting diagnosis. For nursing specifically, there is also a “Time-Critical” alert in the medication administration record (MAR). The purpose of this alert is to convey the importance of administering the PD medication no more than 30 min before or after the scheduled time as opposed to the standard medication administration range of 60 min or greater.

### Throughputs: role development

In our institution, we have developed a voluntary process for nurses to serve as bedside educational support for other nurses. These Nurse Champions act as beside unit resources regarding PD patients to provide real-time and on demand education and support for other nurses. Evidence supports the use of nurse champions to promote best-practice guidelines and close knowledge gaps (18).

### Throughputs: collaborative partnerships

Extensive collaboration was required in order to achieve our desired Outputs. Internally, nurses, providers, and pharmacists working together was crucial since medication management is a focus of care for these patients. Key pharmacy members were identified who helped with assuring PD medications were ordered properly; contraindicated medications were discontinued; adding medication administration instructions and alerts to the EMR; adding PD medications and preparations to our formulary; increasing the number of nursing units with access to the medication; and stocking PD medications in the local medication rooms instead of the central pharmacy for quicker availablity. Our Physical Medicine and Rehab department worked with our patients to address PT, OT, and speech therapy needs.

Evidence supports Nursing care plans to guide clinical practice, (19) therefore, we developed and organized a Parkinson’s Disease Patient Care plan to influence practice change and standardize care for Parkinson’s disease patients. The Care Plan is discussed during multidisciplinary rounds which includes members from the Nursing, Provider, PT/OT, Case management, and Pharmacy Teams. Representatives from this interdisciplinary team met for monthly meetings to share progress and exchange ideas related to PD care. Also, our lead neurosurgeon visited rehab facilities to educate staff and expand our care initiatives to the community as well.

### Throughputs: inquiry

Although there is evidence to direct Parkinson’s care such as the Parkinson’s Foundation “Hospital Care Recommendations,” (4) clear practice guidelines are lacking when compared to other hospital wide diagnoses such as Heart Failure and Diabetes. At our institution, we chose performance measures to prioritize and steer our care. We selected our initial measures and goals when we started our initiative in 2017 based upon environmental scanning. Over time, we went through an ongoing process of developing plans, analyzing outcomes, and revising plans and goals based on our data. Metrics assessing appropriate medication management have been included since the inception of the program. These include ensuring PD medications are ordered using “custom” timing in our EMR to match the patients’ home medication schedules, and monitoring whether contraindicated medications were ordered or administered. In addition to medications, mobility has been another area of focus.

## Outputs/results

The output is evident in the improvement in compliance with our chosen performance measures. We have increased the rate of custom medication ordering nearly three-fold when comparing 2017–2018, (11) and to nearly 50% of orders in 2023 (unpublished yet as of the submission of this article). Furthermore, as a testament to the depth of the education process, we have observed that over half of the orders that were not initially placed as custom are changed to custom by either pharmacy staff or nursing. For the avoidance of contraindicated medications measure, our rate has ranged from 90 to 97% in 2023. Our OOB (Out of Bed) within 24 hours of admission to the unit rate increased from 32% in 2019 to 75% in 2023 (unpublished data).

## Discussion

Evidence supports the role of NPD practitioners in bridging safety gaps with education to improve health outcomes in specific groups of individuals. One of the standards of Nursing Professional Development is health teaching and promotion. NPD practitioners provide education, support, and encouragement for healthcare staff with the overarching goal of promoting population health (14). PD patients are at high risk for development of hospital related complications and are also subject to increased lengths of stay and higher risk of mortality in the hospital. The number of PD patients admitted annually is relatively small compared to other disorders and often they are *scattered* through the hospital based on their admitting diagnoses In order to maintain quality of care in this “high risk, low frequency” patient population, we have utilized the NPD Practice model to introduce initial education and ongoing continuing education with the goal of maintaining competence of inpatient nurses throughout the organization. In this manuscript, we have described the process undertaken by NPD practitioners to support clinical competence in medication management for people admitted to the hospital with PD. Looking to the future, we hope to contribute to the development of practice guidelines to advance the care of Parkinson’s patients admitted to the hospital.

## Data availability statement

The original contributions presented in the study are included in the article/supplementary material, further inquiries can be directed to the corresponding authors.

## Author contributions

MB: Writing – original draft, Writing – review & editing. PP: Writing – original draft, Writing – review & editing. LC: Writing – original draft, Writing – review & editing. HA: Writing – original draft, Writing – review & editing.
